# Fingolimod and tumor-infiltrating lymphocytes in checkpoint-inhibitor treated cancer patients

**DOI:** 10.1007/s00262-020-02693-7

**Published:** 2020-08-17

**Authors:** Omar Hasan Ali, Fiamma Berner, Christoph Jakob Ackermann, Sandra Stephanie Ring, Alexandre Moulin, Joachim Müller, Eva Markert, Oltin Tiberiu Pop, Stefanie Müller, Stefan Diem, Thomas Hundsberger, Lukas Flatz

**Affiliations:** 1 Department of Medical Genetics, Life Sciences Institute, University of British Columbia, Vancouver, Canada; 2 Department of Dermatology, University Hospital Zurich, Zurich, Switzerland; 3 Institute of Immunobiology, Kantonsspital St. Gallen, St. Gallen, Switzerland; 4 Department of Oncology and Hematology, Spital STS AG, Thun, Switzerland; 5 Department of Ophthalmology, L’Hôpital Ophtalmique Jules-Gonin, Lausanne, Switzerland; 6 Department of Nuclear Medicine, Kantonsspital St. Gallen, St. Gallen, Switzerland; 7 Institute of Pathology, Kantonsspital St. Gallen, St. Gallen, Switzerland; 8 Department of Neurology, Kantonsspital St. Gallen, St. Gallen, Switzerland; 9 Department of Oncology and Hematology, Kantonsspital St. Gallen, St. Gallen, Switzerland; 10 Department of Oncology and Hematology, Spital Grabs, Grabs, Switzerland; 11 Department of Dermatology, Venerology and Allergology, Kantonsspital St. Gallen, Rorschacher Strasse 95, 9007 St. Gallen, Switzerland

**Keywords:** Immune checkpoint inhibitors, Oncology, Cancer, Tumor immunology, Fingolimod, Multiple sclerosis

## Abstract

Immune checkpoint inhibitors (ICIs) are emerging as the new standard of care for treating various metastatic cancers. It is known that effective anti-tumor immune responses are associated with a stronger presence of tumor-infiltrating lymphocytes (TILs) in solid tumor tissue. Cancer patients with relapsing–remitting multiple sclerosis (RRMS) are often under continuous treatment with fingolimod, an immune-modulating drug that inhibits lymphocyte egress from secondary lymphatic organs. Little is known about the effect of fingolimod on ICI cancer therapy, as fingolimod may limit the number of TILs. Here we present three patients with RRMS, who developed various cancers during fingolimod treatment. Histology of all tumors consistently showed low numbers of TILs. A second biopsy taken from one of the tumors, a melanoma, revealed a significant increase of TILs after stopping fingolimod and starting pembrolizumab, indicating a surge in the number and re-invigoration of T cells. Our study suggests that fingolimod limits the number of TILs in solid tumors and may, thus, inhibit anti-cancer immune responses.

## Introduction

Immune checkpoint inhibitors (ICIs) have ushered in a new era in the treatment of metastatic cancer. By targeting immune checkpoints, such as the programmed cell death protein 1 (PD1) or its ligand, ICIs lead to durable anti-cancer immune responses [1]. While they had been initially approved for therapy of metastatic melanoma and non-small cell lung cancer, they are now being used for treating various malignancies and have led to significant improvement of clinical outcomes and quality of life [2, 3]. However, not all patients show therapy response and validated predictive biomarkers are scarce. One of the few reproducible markers is the proportion of tumor-infiltrating lymphocytes (TILs): a more pronounced pre-treatment lymphocytic infiltrate in solid tumors is associated with a better ICI therapy response [4]. Multiple sclerosis (MS) is a chronic inflammatory demyelinating disease of the central nervous system (CNS) caused by auto-reactive T cells that migrate into the brain, where they induce inflammation and functional impairment. While the disease may be of multifactorial origin its exact mechanisms of the disease remain unknown [5]. For relapsing–remitting MS (RRMS) fingolimod has been established as a golden standard of care. Fingolimod is an active sphingosine 1-phosphate receptor (S1PR) antagonist that is orally administered. S1PRs are present on the surface of various cells including lymphocytes and neurons. Under normal circumstances activation of S1PRs overrides inhibitory signals from the homing C–C chemokine receptor 7, present mainly on naïve and central memory (CM) T cells, allowing these cells to egress from lymph nodes. However, in the presence of fingolimod, S1PRs are internalized and degraded, leading to the selective retention of naïve and CM T cells in secondary lymphoid organs and reducing the migration of lymphocytes to the CNS. In addition to its selective effects on the immune system, fingolimod is also thought to exert direct effects in the CNS, mainly by promoting oligodendrocyte-mediated remyelination and by reducing leakage of the blood–brain barrier [6].

The clinical introduction of fingolimod has marked an evolutionary step in the treatment of MS. However, its property to inhibit lymphocyte egress may negatively affect immune responses against cancer. Large pivotal phase III studies (FREEDOMS, FREEDOMS II, and TRANSFORMS) for Gilenya® (fingolimod) have shown no increased risk of developing neoplasms during therapy [7]. However, post-marketing surveillance studies and numerous case reports suggest that the risk for developing cancers during treatment may be underestimated, especially for melanoma and cutaneous lymphomas [8–11]. The use of fingolimod is particularly challenging in case of patients with newly diagnosed MS with a medical history of neoplasms. Here we present three patients, of whom one experienced a relapse of her melanoma and two developed new cancers during fingolimod treatment.

## Methods

Clinical data and positron emission tomography/computed tomography scans were collected from three cancer patients with RRMS who were under fingolimod treatment during first cancer diagnosis. All patients were treated in the Department of Oncology and Hematology of the Kantonsspital St. Gallen. Tumor samples that had been obtained for diagnostic histological examination were formalin-fixed and paraffin-embedded (FFPE) and stained with hematoxylin and eosin in the Institute of Pathology of the Kantonsspital St. Gallen using the standard protocols. Single epitope enzymatic immunohistochemistry on FFPE tissue was performed on four-micron thick serial sections using a Leica BOND MAX automated immunostainer and the following antibodies: monoclonal mouse anti-human CD3 (Novocastra Biosystems, clone LN10, catalog number NCL-L-CD3-565, dilution 1:120, HIER—pH 9/30 min/100 °C, incubation for 30 min), monoclonal mouse anti-human CD4 (Novocastra Biosystems, clone 4B12, Catalog No. NCL-CD4-368, dilution 1:60, HIER—pH 9/30 min/100 °C, incubation for 30 min), and monoclonal mouse anti-human CD8 (Dako/Agilent, clone C8/144B, catalog number M7103, dilution 1:120, HIER—pH 9/20 min/100 °C, incubation for 15 min). Micrographs were acquired with a Leica DM RA microscope equipped with a Leica DFC420 C digital camera and processed using the Leica Application Suite version 3.8.0 (Leica Microsystems, Switzerland), followed by assessment of TILs.

## Results

**Fig. 1:**
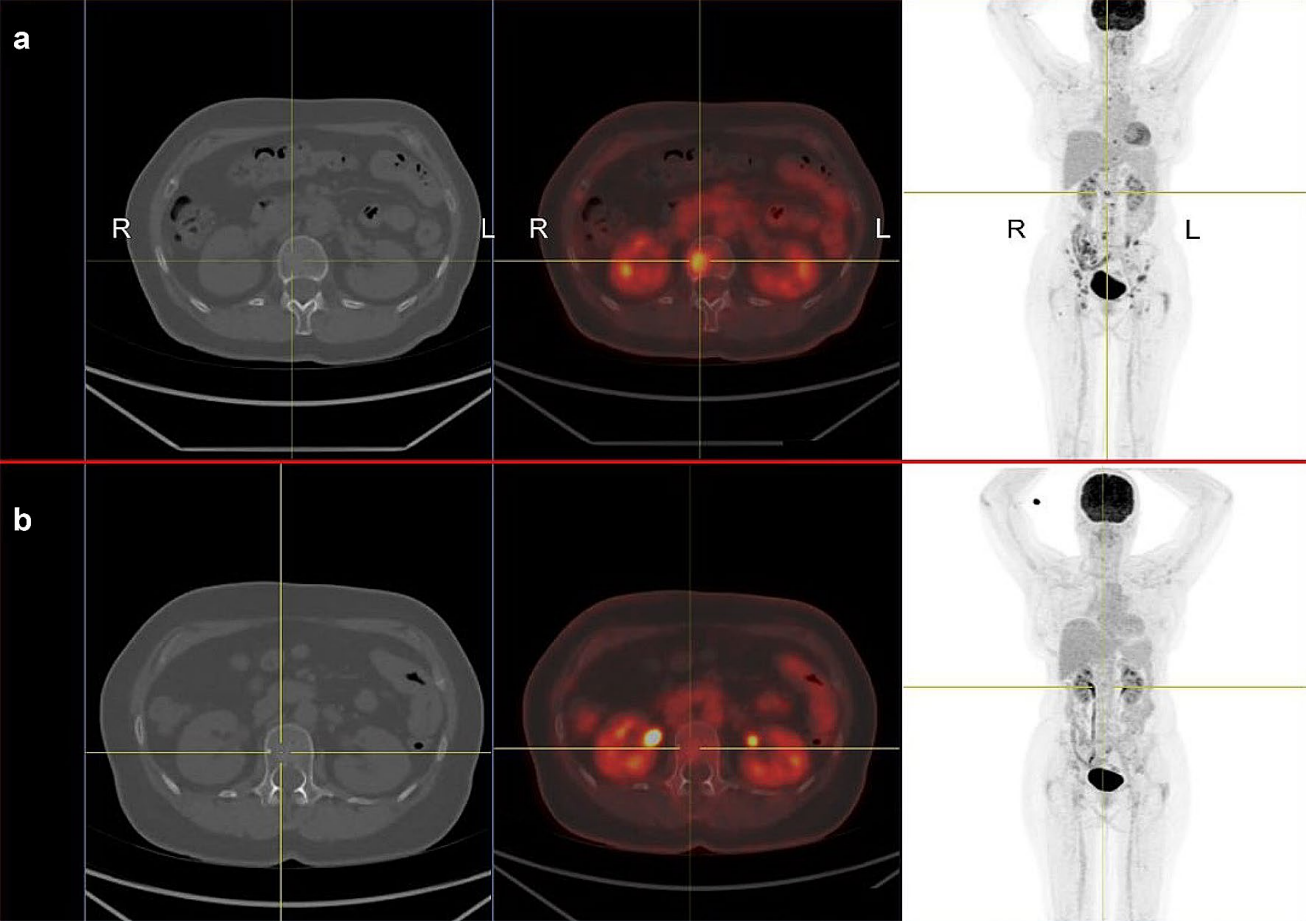
Relapsing metastatic melanoma and response to anti-PD1 treatment in a patient with fingolimod. **a** 18F-FDG PET/CT scan shows multiple bone metastases of melanoma, including the spine (centered by yellow crosshair). The patient has relapsing-remittent multiple sclerosis (RRMS) and is under treatment with fingolimod. *R* right, *L* left. **b** After ceasing fingolimod and starting treatment with pembrolizumab the follow-up PET/CT scan after 12 weeks shows a partial remission of all bone metastases including the spine. To date the patient showed no flares of RRMS, despite complete cessation of fingolimod

**Fig. 2:**
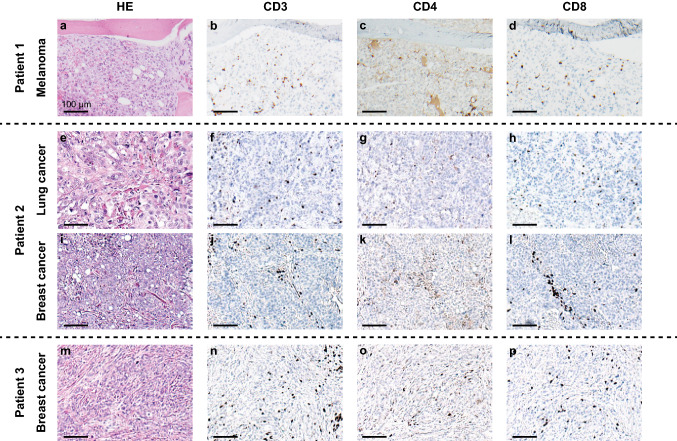
Tumors of fingolimod-treated patients have low numbers of tumor-infiltrating lymphocytes. Representative micrographs of tumor samples stained with hematoxylin and eosin (left column) and single epitope immunohistochemistry for CD3 (center-left column) CD4 (center-right column) and CD8 (right column). **a**–**d** Patient 1—melanoma. **e**–**h** Patient 2—lung cancer. **i**–**l** Patient 2—breast cancer. **m**–**p** Patient 3—breast cancer. Scale bars = 100 µm

**Fig. 3:**
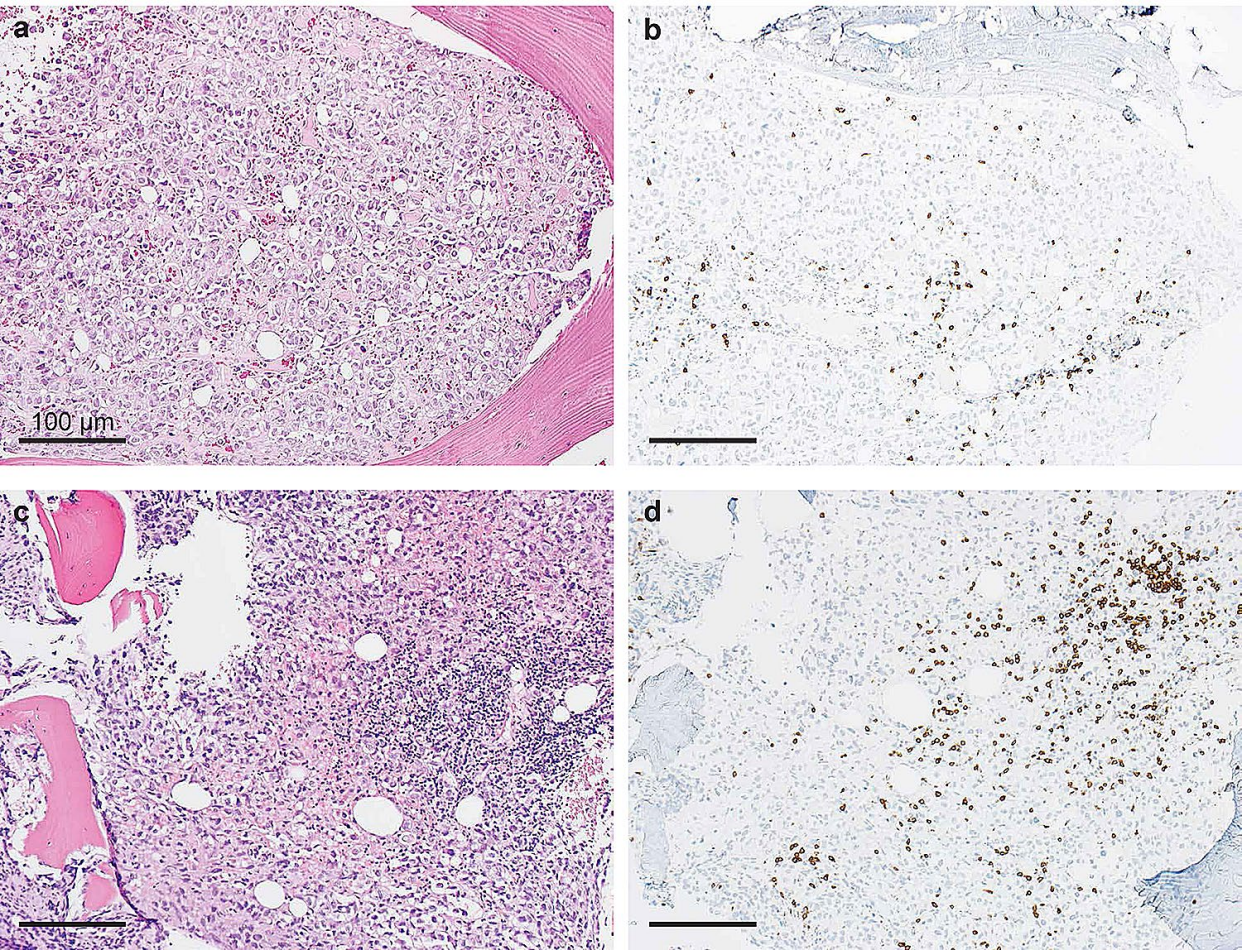
Increase in tumor-infiltrating lymphocytes (TILs) in melanoma after cessation of fingolimod and initiation of pembrolizumab therapy. **a**, **b** Representative micrographs of metastatic melanoma biopsy from patient 1 during fingolimod therapy and before initiation of pembrolizumab: the tumor shows sparse lymphocytic infiltrate. **a** Hematoxylin and eosin (HE), **b** single epitope immunohistochemistry (IHC) for CD3. **c**, **d** Tumor biopsy from the same patient after cessation of fingolimod and three cycles of pembrolizumab: both HE (**c**) and IHC for CD3 (**d**) show marked increase in TILs

*Patient 1* A 61-year old woman was diagnosed with uveal melanoma in March 2012, which was treated with proton beam therapy, which lead to complete tumor remission. After several years of asymptomatic follow-up examinations in our oncological department she reported to have increasing back pain that did not subside during periods of resting (Fig. 1a). A PET-CT scan in November 2016 showed metastatic cancer with extensive spread to several bones, including the spine. Tumor biopsy confirmed metastatic melanoma. The patient also suffered from RRMS and has been on treatment with fingolimod since 2013. Due to new metastatic disease fingolimod was stopped in January 2017. Despite the risk of MS flare-up during ICI treatment she received anti-PD1 treatment with pembrolizumab in January 2017, which showed excellent response (Fig. 1b). After an initial response, she developed new bone metastases in July 2017 and was switched to anti-CTLA4 treatment with ipilimumab. The first biopsy was obtained during fingolimod treatment and revealed very sparse lymphocytic infiltrate, as shown in Fig. 2a–d and in Fig. 3a, b. Interestingly, a follow-up biopsy taken after stopping fingolimod and during pembrolizumab treatment showed significantly more lymphocytic infiltration compared to the first biopsy (Fig. 3c, d), indicating immune system invigoration against the tumor. The patient showed good therapy response to ipilimumab and has not experienced any MS flares during or after therapy.

*Patient 2* A 51-year old women was admitted to the emergency department due to dyspnea and chest pain in March 2016. A thorax CT scan revealed pulmonary embolism and a tumor in the right lung. Histology of tumor tissue revealed adenocarcinoma of the lung. While the subsequent PET/CT scan showed no signs of distant metastases, it displayed increase contrast uptake at a lump in the left breast. Biopsy was performed and histology revealed intraductal breast cancer. The patient had been under treatment with fingolimod for RRMS since September 2015, which was stopped after tumor diagnosis. Similarly to patient 1, histology of both tumors showed a very sparse lymphocytic infiltrate in tumor tissue, as displayed in Fig. 2e–l.

*Patient 3* A 51-year old woman was admitted to the gynecology department due to a palpable lump in her left breast in June 2014 and histology confirmed intraductal breast cancer. She had been treated with fingolimod for RRMS since July 2012. Fingolimod was paused and the patient underwent complete resection of the tumor, sentinel lymph node biopsy and adjuvant chemo- and radiotherapy. After tumor clearance fingolimod was resumed. Also in her case, histology showed an almost total absence of lymphocytic infiltration (Fig. 2m–p).

## Discussion

The management of patients with metastatic cancer and RRMS under fingolimod presents a special clinical challenge, as re-invigoration of T cells may induce flare-up of MS. In most phase III studies investigating ICI based treatments patients with pre-existing autoimmune diseases have typically been excluded. Indeed, there are reports of MS flares during ICI treatment [12]. A recent meta-analysis found that flares of MS during ICI treatment are rare, but severe [13]. To date, in our case, neither pembrolizumab nor ipilimumb lead to RRMS flares, despite cessation of treatment with fingolimod, while effectively treating cancer. In the case of metastatic cancer, such as in our patient, the argument for ICI therapy is evident as it outweighs the risk of autoimmune adverse events. For adjuvant ICI therapy however, a careful waging of potential risk and benefit is necessary to make the most appropriate choice on a case-by-case basis.

Following the examination of tumor samples from cancer patients with MS, we found a potential association between the use of fingolimod and reduced numbers of TILs, regardless of the type of cancer. This may be explained by the mechanism of fingolimod, which prevents lymphocyte egress from secondary lymphoid organs. Pre-existing TILs in solid tumors have been shown to be an important biomarker in predicting response to ICI therapy [4, 14]. Given that ICIs invigorate exhausted effector T cells [15], their presence in solid tumors indicates an effective anti-tumor response. Additionally, TILs have been shown to be crucial for better clinical outcome in cancer patients treated with other cancer drugs, such as chemotherapy [16]. Thus, the presence of TILs in solid tumors suggests effective anti-tumor activity, regardless of the given anti-cancer drug type. In our first patient, tumor histology during fingolimod treatment showed a remarkably less pronounced lymphocytic infiltrate compared to tumor tissue taken after cessation of fingolimod. This potential TIL restriction of fingolimod is supported by a previous study, which found that fingolimod reduced the recirculation of CD8^+^ effector T cells and their recruitment to peripheral lesions in a mouse model of diabetes [17]. In another study the authors were able to demonstrate that fingolimod inhibits anti-tumor immunity, leading to the development of myeloma and B-cell lymphoma [18]. On the other hand, cancer studies on mouse models suggest that fingolimod may have a sensitizing effect on certain cancer pathways and may increase the efficacy of chemo- or radiotherapy in some cancer types [19, 20]. These data highlight the urgent necessity of additional studies to investigate the impact of fingolimod on anti-tumor immune responses to assist physicians in making informed decisions for choosing the most appropriate MS therapy.

In summary, we present a patient with relapsing metastatic melanoma and RRMS, who responded to ICI therapy and experienced no flare-up of RRMS despite stopping of fingolimod treatment. This argues for ICI safety during MS therapy. Furthermore, we found that fingolimod may be associated with lower TIL numbers in solid organs, suggesting a possibly impaired anti-tumor response during treatment. Additional large-scale studies and mechanistic explorations are necessary to substantiate these findings.

## Abbreviations

CM: Central memory
CNS: Central nervous system
FFPE: Formalin-fixed paraffin embedded
HE: Hematoxylin and eosin
ICIs: Immune checkpoint inhibitors
IHC: Immunohistochemistry
MS: Multiple sclerosis
PD1: Programmed cell death protein 1
RRMS: Relapsing–remitting multiple sclerosis
S1PRs: Sphingosine 1-phosphate receptor
TILs: Tumor-infiltrating lymphocytes
